# Impact of the COVID-19 pandemic and the related containment measures on the mental health of children and adolescents

**DOI:** 10.25646/7174

**Published:** 2020-12-09

**Authors:** Robert Schlack, Laura Neuperdt, Heike Hölling, Freia De Bock, Ulrike Ravens-Sieberer, Elvira Mauz, Benjamin Wachtler, Ann-Kristin Beyer

**Affiliations:** 1 Robert Koch Institute, Berlin Department of Epidemiology and Health Monitoring; 2 Federal Centre for Health Education, Cologne Department Effectiveness and Efficiency of Health Education; 3 University Medical Center Hamburg-Eppendorf Center for Psychosocial Medicine, Clinic for Child and Adolescent Psychiatry, Psychotherapy and Psychosomatics, Child Public Health

**Keywords:** COVID-19, MENTAL HEALTH, CHILDREN, ADOLESCENTS, SCHOOL AND NURSERY CLOSURES, CHILD PROTECTION

## Abstract

Children and adolescents are particularly affected by the COVID-19 pandemic and the official containment measures. However, the effects on their mental health have been little studied. The aim of this narrative review is to summarize existing evidence on the mental health of children and adolescents in the first weeks of the COVID-19 pandemic and during the measures taken to contain it in Germany. First international and national studies draw a differential picture. Children and adolescents showed symptoms of anxiety and depression as well as a reduced quality of life. The closure of childcare and educational facilities and the associated loss of the familiar daytime structure as well as loss of contact and independent learning at home posed considerable challenges for affected children and their families. Spatial confinement at home and the lack of alternative options of stay during the containment measures could also have lead to increased family stress, heightened family aggression, and domestic violence. However, the findings of several studies also show that many families coped with the time during the containment measures mostly well. In the event of possible future pandemics or further waves of the COVID-19 pandemic, the needs of adolescents and their families during the containment measures should be given greater consideration.

## 1. Introduction

On March 11, 2020, the World Health Organization (WHO) officially declared the outbreak of the Severe Acute Respiratory Syndrome Coronavirus 2 (SARS-CoV-2) as pandemic against the background of its increasingly global spread [1]. In the wake of this decision, the populations of entire countries have faced various non-pharmaceutical containment measures such as restrictions on social contact, school and nursery closures, and social distancing and quarantining measures that have drastically altered people’s everyday lives. Soon, there was a call to take into account the implications of these measures for the mental health of the population [2]. In accordance with WHO recommendations, several rapid reviews were published in quick succession [3]. The reviews summarized relevant results from previous outbreaks and, above all, the empirical work conducted in China on ‘Coronavirus Disease 2019’ (COVID-19) [4–6]. The situation of children and adolescents during the pandemic, as well as the impact of the containment measures in Germany, have received little attention so far. Nevertheless, as a developmentally vulnerable population group, children and adolescents are particularly affected by the effects of the pandemic and the related containment measures [7, 8]. This review focuses on three areas that can be viewed as particularly relevant to the mental health of children and adolescents in the first weeks during the COVID-19 pandemic: 1) the impact of the pandemic and the associated containment measures on the mental health and wellbeing of children and adolescents; 2) the importance of the closure of educational and day-care facilities; and, 3) family tensions, domestic violence and child protection issues linked to containment measures.

## 2. Methodology

This study was conducted as a narrative review so as to provide an initial overview as quickly as possible and to ensure that the results presented here are highly relevant to the current situation. The findings are based on research conducted between March and 20 May 2020. A search was undertaken of PubMed’s LitCovid literature database using the keywords ‘Psych’, ‘Psyche’, ‘Mental’, and ‘Trauma’, and relevant papers about children and adolescents were selected. Further studies were identified ad-hoc using the PubMed and Google Scholar literature databases in combination with the search terms ‘COVID-19’ AND ‘pandemic’, ‘child*’, ‘adolescent*’, ‘mental health’, ‘school *closure’, ‘home schooling’, ‘preschool’, ‘kindergarten’, ‘disaster’, ‘domestic violence’, and ‘child abuse and neglect’. The search focused on literature in English or German language about the current pandemic and also about previous epidemics and pandemics. Articles were selected if they addressed the impact of the current pandemic on childrens' and adolescents' mental health, the closure of educational or day-care facilities, family tensions, domestic violence or child protection issues. Furthermore, preprints (preliminary publications that have yet to undergo peer review), reports in the press and reports found using the Google search engine were identified between March and early July 2020 in order to include initial empirical findings. Publications that appeared after the end of the search period could not be considered. The selection of the literature was subjective and unsystematic, and, therefore, no claims are made about completeness or reproducibility.

## 3. Results

### 3.1 Mental health and wellbeing

Current reviews indicate that children and adolescents frequently developed symptoms of depression and anxiety in the first weeks during the COVID-19 pandemic [8–10]. According to an online survey conducted in 21 provinces and autonomous regions in China of 8,140 school pupils aged from 12 to 18, 43.7% of respondents displayed symptoms of depression and 37.4% showed symptoms of anxiety during the COVID-19 outbreak [11]. The most common depressive symptoms identified included displaying little interest or pleasure in doing things (53.9%), feeling tired or having little energy (48.4%), and either a poor appetite or overeating (45.6%). The most frequent symptoms of anxiety included feeling nervous, anxious or on edge (53.6%), worrying too much (47.3%), and becoming easily annoyed or irritable (47.0%). The nationally representative German COPSY study (impact of **CO**VID-19 on **psy**chological health in children and adolescents) surveyed 1,040 children and adolescents in Germany between the ages of 11 and 17 as well as 1,586 parents of 7- to 17-year-olds in May and June 2020 about the effects of the COVID-19 pandemic on the mental health, quality of life and health-related behaviour of children and adolescents. Overall, 40.2% of the 11- to 17-year-olds surveyed reported a decrease in their quality of life, whilst 31.0% of the 7- to 17-year-olds showed psychological problems [12]. Comparative data from the population-based study of the behaviour and well-being of children and adolescents in Germany (BELLA study) [13] indicates that before the pandemic only 33.0% of participants had been affected by a reduced quality of life and 18.0% by mental health problems. Similarly, the frequency of symptoms of anxiety increased from 15.0% to 24.0% during the pandemic [12]. Results of a study carried out across Germany at the beginning of May 2020 on behalf of the statutory health insurance company DAK Gesundheit with 1,005 parents and their children (aged between 10 and 17) found that 18.0% of children and adolescents had frequent concerns about the impact of the pandemic. Their concerns related to issues such as societal development, school and the economic consequences of the pandemic. At the same time, 19.0% of children and adolescents were worried about contracting COVID-19 or that a loved one might do so [14].

Families also reported increased levels of stress, which, among other things, were caused by the fact that many families had to reorganize their everyday life during the closures of educational and childcare facilities and parents had to work in the home office or reorganize their work outside the home in parallel. In some cases, existential concerns about limited earning opportunities, short-time work or job loss were added to the mix. Especially in younger children, increased family stress levels can be expressed by regressive behaviour such as crying or a relapse into non-age-appropriate behaviour, but also irritability and aggressiveness [15]. In addition, children and adolescents may develop emotional (e.g. excessive worry or sadness) and psychosomatic symptoms (e.g. unexplained headaches or physical pain) [16]. Simultanoeously, children and adolescents had fewer opportunities to regulate their stress (such as by letting off steam in playgrounds and meet their peers) [8]. Children who were quarantined in hospitals or medical observation centres, experienced sadness and anxiety due to being separated from their parents and were more vulnerable regarding mental health problems [10].

Furthermore, the pandemic and the containment measures may also worsen the symptoms in children and adolescents with preexisting mental disorders [17]. For children and adolescents with attention-deficit/hyperactivity disorder (ADHD), for example, the loss of daily structure and extra-familial social interaction can exacerbate the symptoms of the disorder [18]. In addition, symptoms may worsen if pharmacological treatment is discontinued due to the pandemic, since the availability of medication is of great importance for ADHD patients [18, 19].

### 3.2 Closures of educational and day-care facilities

Data from the United Nations Educational, Scientific and Cultural Organization (UNESCO) show that by the time educational and day-care facilities were closed throughout Germany on 18 March 2020, schools had already been closed in 126 countries. In the first half of April 2020, around 1,580,000,000 children and adolescents (around 90% of school children) in 194 countries were affected by school closures [20].

The closure of educational and day-care facilities for several weeks, in addition to other containment measures, poses challenges to and makes it more difficult for children and their families to cope with everyday life [21]. In addition to the demands of the job or working in a home office, there were also increased family necessities in terms of child care or schooling for the parents. In addition, emotional demands increased because of the need to explain the pandemic and its consequences to the children as calmly and safely as possible, to provide security and to counteract fear or worries in the family [8]. At the same time, the support of grandparents was often lacking, as they are at risk of developing COVID-19 disease due to their age. There have been several public recommendations from key political actors to avoid contact with grandparents during the containment phase [22]. In a survey conducted as part of the COVID-19 Snapshot Monitoring (COSMO study) at the University of Erfurt, in which the Federal Centre for Health Education (BZgA) and the Robert Koch Institute (RKI) are also involved, parents found it most distressing that their children could no longer see their grandparents. They also saw other challenges in encouraging the children, as well as organizing their education and work [23].

The German Youth Institute conducted a study to analyse the ways in which the COVID-19 pandemic had changed people’s everyday lives. It surveyed over 8,000 parents of 3- to 15-year-old children and adolescents. The majority reported having dealt with the challenges fairly or very well. A third of parents stated that their children have had difficulties coping with the current situation [21]. DAK Gesundheit’s study involving parents and their children found that 31.0% of children had often or very often experienced emotional stress during the pandemic-related school closures. 24.0% of children reported frequent or very frequent arguments in the family, and 25.0% described having feelings of sadness [14]. Results from the COPSY study suggest that two-thirds of children and adolescents have found school and learning to be more strenuous during the pandemic [24]. Children from families with low socioeconomic status (SES) or with a migrant background were particularly affected by the negative effects of the pandemic on learning. For example, they often have only limited technical facilities for independent learning at home. In addition, cramped living conditions could make it difficult for them to complete their schoolwork undisturbed. In Germany, especially in large cities, housing is distributed to the disadvantage of people with low incomes [25]. This can contribute to the fact that children from socially disadvantaged families in particular have problems in coping with everyday school life while learning independently at home [12]. In families with a migrant background, parental support for independent learning at home could be difficult due to language barriers [26].

Moreover, day-care centres and schools not only provide care and education to young people, but also offer them opportunities to meet friends and peers, engage in leisure activities and to have a warm meal; the latter may be particularly important for children from families with a low SES. In addition, schools and day-care centres also enable children the access to health promotion and disease prevention [27], and this has been lacking since the closure of schools and day-care centres.

### 3.3 Family tensions, domestic violence and child protection

Spatial confinement at home during the containment measures, coupled with other already mentioned pandemic-related stressors such as loss of the accustomed daily structure or job insecurity, could promote latent conflict potential in the family and lead to an increase in intra-family aggression and disputes [28]. However, results from five waves of the COSMO survey by the University of Erfurt found in total no significant increase in the number of arguments between parents during spring 2020 [23]. Nevertheless, when the age of the children is considered, families with younger children (under 14 years of age) faced a significantly higher burden than families with adolescents (14 years of age and older). Over time, however, in families with younger children, the feeling of strain gradually approached the level of families without younger children in the household, possibly as a result of the relaxation of containment measures [23, 29] and/or adaptation to the situation. Results from the COPSY study suggest that the pandemic worsened the mood in families: 27.0% of the children and adolescents and 37.0% of the parents reported that the frequency of arguments had increased [12].

In addition to intra-family aggression and disputes, the pandemic and the associated containment measures could also increase the risk of manifest domestic violence and child abuse or aggravate existing domestic violence [30, 31]. Experiences of violence are one of the strongest risk factors for the mental health of children and adolescents [32] and are associated with potential mental health consequences that could last into adulthood [33, 34]. Children may witness domestic violence between their parents or caregivers or be directly affected themselves [35]. As a result of the containment measures, an increase in alcohol abuse is expected [36]. This is another potential risk factor for domestic violence and child abuse during the pandemic [37]. However, the results of the COSMO survey did not show a trend towards more frequent alcohol consumption in Germany [38].

Nevertheless, reports from around the world, primarily published in the press, indicate that services for people affected by domestic violence are being used more frequently than before the pandemic [39]. A press release from the German Federal Ministry for Family Affairs, Senior Citizens, Women and Youth states that the number of calls to the Germany-wide ‘Violence against women’ helpline rose in mid-April by 17.5% over the previous two weeks [40]. A representative survey of 3,800 women in Germany by the Technical University of Munich on experiences of violence found that children faced physical punishment in 6.5% of households [41]. This study also identified a greater frequency of verbal abuse and physical violence as well as violence against young people in households with children under ten years of age [41]. However, press releases either demonstrated no increase or just a slight increase in the number of crimes of this nature being reported, despite an increase in police call-outs for domestic violence during the pandemic [42].

Increases in neglect, child abuse and sexual violence against children and adolescents have been observed worldwide during previous social crises and pandemics [43–45]. Even if there are no reliable data for Germany, restrictions during spring 2020 can be assumed to have led children to face a greater risk of physical and sexual violence, and this especially applies to children with intellectual or physical disabilities and those in families that are facing particularly high levels of stress [7, 8].

## 4. Discussion

The aim of this narrative review was to summarize existing evidence on the impact of the COVID-19 pandemic and the measures taken to contain it on the mental health of children and adolescents. Children and adolescents are among the population groups that have received little attention in the COVID-19 pandemic.

In particular, symptoms of anxiety and depression are more common in children and adolescents during the COVID-19 pandemic [8–10, 12, 14]. Children and adolescents with pre-existing mental disorders such as ADHD are a particularly vulnerable group [18, 19]. During a crisis situation, there is an increased need for bonding with parents [46], which is why separation due to quarantine outside the home or due to a hospital stay of one parent is perceived by the children as very stressful [47]. The pandemic-related closures of educational and care facilities and the associated loss of the familiar daily structure, contact breaks with persons with whom the children have ties outside the home, as well as parallel learning at home and work by the parents, posed considerable challenges for all. First data suggest, however, that many families coped with this situation largely well. For some, however, it also represented a high burden [21, 48], especially for families with younger children [23]. Children from families with low SES or with a migrant background may also be more affected by school closures [26, 49], as they may be more vulnerable to the COVID-19 pandemic, particularly in terms of educational opportunities. As a result, social inequalities that already existed before the pandemic could further increase.

A lack of space and other alternatives are risk factors associated with stress and aggression in families, and with domestic violence [23, 28]. This association increases in households with younger children [23]. In addition, containment measures also lead to a greater risk of child-rearing practices that involve physical discipline [7, 41]. Therefore, it is likely that the risk of neglect, physical abuse and child abuse has increased since the implementation of the containment measures [7]. Nevertheless, Germany has seen a reduction in the number of reported child protection cases during this period compared with before the pandemic [50]. This trend has also been observed in other European countries [51]. However, this may probably be due to the temporary closure of educational and day-care facilities, youth welfare offices and other youth welfare agencies. This view is supported by the German Child Protection Association’s statement on the ‘Situation of children and adolescents in the Corona crisis’, which was submitted to the children’s commission in the German Bundestag. The statement emphasises that before the pandemic 60% of all child protection reports were filed by schools, day-care centres and paediatric practices [52]. In any event, inquiries from child and adolescent psychotherapeutic, paediatric and child and adolescent psychiatric clinics to the nationwide medical child protection hotline have increased since the beginning of the pandemic [7].

This review is limited by the current paucity of empirical evidence on the impact of the pandemic on the mental health of children and adolescents in Germany. The extent to which the results from countries with different social systems and possibly different cultural characteristics can be transferred to the situation in Germany cannot be conclusively answered here either. However, a comparison of the results from China presented above with those from Germany suggest that the pandemic is having a similar impact on children’s mental health there and here.

Children and adolescents are a largely overlooked group in the social debate about the consequences of the pandemic and the measures to contain it. According to the initial results for Germany, most families appear to have been reasonably successful in meeting the challenges that this entails. However, the results summarized here suggest that the mental health and well-being of children and adolescents may have deteriorated under the conditions of the pandemic and the containment measures. Further studies are needed to provide more details about the preliminary results presented here, especially in terms of the impact on vulnerable risk groups. This is essential if preventive measures are to be drawn up for future pandemics and COVID-19 waves. Finally, the results indicate that families with lower SES, with younger children, those with children with intellectual or physical disabilities or pre-existing mental illnesses and disorders need target group-specific measures to reduce the risk of stress, excessive demands, conflicts and domestic violence in the families [24, 49].

## Key statements

- Initial studies show that the COVID-19 pandemic and the related disease containment measures have led to increased symptoms of anxiety and depression among children and adolescents and reduced their quality of life.
- Children and adolescents found school and learning to be more strenuous. Children from socially disadvantaged families and children and adolescents with a migration background have had the most problems coping with learning at home.
- A lack of space at home and other places to go during the containment measures can put a greater burden on families, lead to increased levels of aggression between family members, and exacerbate the risk of domestic violence and incidents involving child protection issues.
- First data suggest that many families coped relatively well during the pandemic and the containment measures.
